# Language and autobiographical memory development from 5 to 12 years: A longitudinal perspective

**DOI:** 10.3758/s13421-024-01544-5

**Published:** 2024-03-05

**Authors:** Llanos Merín, Alonso Mateo, Marta Nieto, Laura Ros, José Miguel Latorre

**Affiliations:** 1 Department of Psychology, Faculty of Medicine, University of Castilla, La Mancha, Avenida de Almansa 14, 02006 Albacete, Spain; 2 Applied Cognitive Psychology Unit University of Castilla-La Mancha, Albacete, Spain; 3 Faculty of Education, University of Castilla-La Mancha, Albacete, Spain

**Keywords:** Autobiographical memory, Memory specificity, Verbal ability, Longitudinal perspective

## Abstract

The main aim of this study, with two repeated measurements, was to analyze the development of autobiographical memory in a sample of 78 Spanish participants at ages 5 (Time 1; *M* = 62.43 months, range: 50–74 months) and 12 (Time 2; *M* = 142.71 months, range: 132–155 months). Data were collected on autobiographical memory and verbal functions. We analyzed the relation between language and autobiographical memory specificity from a longitudinal perspective and assessed the indirect effect of vocabulary in the relationship between age and specific memory at both temporal moments. The results showed that language skills were positively related with autobiographical memory specificity at preschool age, but not at the second measurement. Furthermore, vocabulary scores appear to mediate the relationship between age and autobiographical specificity when children are in the preschool years, but not later. These findings agree with previous research that consider preschool age to be a crucial period for the development of autobiographical memory and its relations with language, but once basic command of language is acquired, linguistic differences impact much less on individual differences in autobiographical specificity.

Autobiographical Memory (AM) refers to a memory storage system for personal information that includes episodic memories and more conceptual self-related information (Conway & Pleydell-Pearce, 2000). Although it has been generally considered as a case of explicit memory, developmental researchers have differentiated it from other interrelated memory systems such as episodic memory (e.g., Bauer, 2015; Howe, 2014). According to Courage and Howe (2022), *episodic memory* refers to the recall of event information that is associated with a particular time, place or person but that is not personally relevant; however, when episodic memories include relevant personal information, and are associated with ones’ self, it would form part of *autobiographical memory*. Therefore, AM differs from episodic memory essentially in the personal, subjective, or evaluative perspective included in the memory of the event (Courage & Howe, 2022). Thus, AM allows us to remember experiences that happened to ourselves, including information about the who, what, where, when, why, and how of the personal events (Nelson & Fivush, 2020).

The AM system is hierarchically organized in different levels of specificity (Conway & Pleydell-Pearce, 2000). The top level consists of *extended memories*, which are general memories which recall general events lasting more than one day (e.g., “the week I spent at a camp with my friends”) and *categorical memories*, which refer to repeated events grouped into categories (e.g., “Christmas dinners with my family”). At the bottom of the AM hierarchy are located *specific memories*, which are personally significant memories associated with a concrete moment and place and that last less than 24 hours (e.g., “the day I passed my driving test”).

In recent years there has been an increase in the literature on the characteristics and implications of AM in different contexts (e.g., clinical, educational) in children and adults (e.g., Brien et al., 2021; Fivush & Grysman, 2022; Ros et al., 2017) and, to a lesser extent, on the origins and evolution of AM in early childhood (e.g., Nieto et al., 2018; Nuttall et al., 2014; Wang, 2008). Additionally, longitudinal research has reported mixed results on the role of AM specificity in the risk of psychopathology during childhood and adolescence (i.e., Gutenbrunner et al., 2019; Warne et al., 2020), or about the predictors of the age-related and individual variability found in AM in childhood and adolescence (Bauer & Larkina, 2020; Edler et al., 2021). However, to our knowledge, there is no research that examines the evolution of AM from preschool age to early adolescence. Moreover, despite the advances in autobiographical studies in the past years, the link between children’s language skills and AM is less well established (Bartoli & Smorti, 2019). Therefore, our aims are to analyze the progress of AMs from a longitudinal perspective in the same sample of participants at 5 and 12 years of age and to establish the role of language in the development of AM specificity.

## Development of autobiographical memory and the role of language

The ability to form AMs is a developmental achievement of childhood that is influenced by several factors: (1) relevance and emotional valence of the event (e.g., Luminet, 2022; Occhionero et al., 2023); (2) language development, since it allows individuals to organize, situate and construct personal experience in coherent narratives (e.g., Fivush, 2019; Nelson & Fivush, 2020); (3) the maturation of central nervous system (CNS), which allows the development of cognitive abilities, such as executive functions, necessary for the encoding, maintenance and retrieval of memories (e.g., Nieto et al., 2018; Piolino et al., 2002); and (4) the emergence of self-consciousness, or a cognitive sense of self (e.g., Courage & Howe, 2022; Ross et al., 2020). This paper will focus on the role of language in the development and consolidation of the AM. Even if AM per se does not necessarily need language to be perceived (as it can also be made of smells, colours, images, body perceptions, sounds), most of the memories require language to be organized and shared with others in a specific social and cultural environment (Bartoli & Smorti, 2019; Nelson & Fivush, 2020).

The first signs of the development of AM appear around age 2, with the use of personal pronouns and the ability to recognize oneself in a mirror (Markowitsch & Welzer, 2010). Infants’ growing memory capacities can also be expressed through language: Ss soon as they are able to combine two meaningful words together (at about 16–18 months), they begin to spontaneously refer to the past (Fivush, 2011). However, it is not possible to speak of a functional level of AM until the preschool years. This period, form about 3 to 6 years of age, is a critical time for the development of certain capacities that depend on brain maturation, such as language, narrative skills, memory abilities, self-awareness, and executive functions (Howe, 2014; Nieto et al., 2018; Ross et al., 2020). At about age 3, children begin to develop relatively coherent narratives about their past experiences, particularly when such narratives are produced in the setting of interactions with parents (Leyva et al., 2020). These memories usually refer to relatively recent experiences and are completed by the adults, who take the verbal references provided by the child and put them into a more coherent narrative form (Fivush, 2011). Memory is a social activity and acquires personal meaning through shared experiences. Hence, the quality of adult reminiscing style and the cultural context in which a child develops have an influence on AM (Fivush, 2019; Nelson & Fivush, 2020; Wang, 2021; Waters et al., 2019). Individual differences in the way parents, and specifically mothers, structure verbal reminiscing with their children are usually linked to individual differences in children’s verbal productions and developing AMs (Fivush, 2011). Indeed, mothers’ style of reminiscing is an established contributor to AM, both as memories are forming in early childhood and later when memories are retrieved in adolescence (see Wu & Jobson, 2019, for a review).

Focusing on the ability to retrieve specific AMs, it has been suggested children represent and retrieve general event memories from an early age and, as they develop, they acquire a more specific style (Bauer, 2015; Williams, 1996). More concretely, Bruce et al. (2000) proposed that AM specificity begins to emerge from three years and consolidates from the age of 4 ½ years. Cross-sectional studies with preschoolers have found that AM specificity is positively associated with age (Nieto et al., 2017, 2018; Nuttall et al., 2014). During preschool years, brain maturation leads to the progressive development of different cognitive skills, including memory and language skills (Courage & Howe, 2022). The emergence of their ability to encode, store, and retrieve information using a linguistic code underlies the changes that occur in memory (Simcock & Hayne, 2003). Moreover, language facilitates the retrieval of information through a better understanding of the event that is intended to be remembered. For instance, Wang (2008) showed that preschoolers’ with stronger language skills developed a better understanding of emotions, which in turn helped them to develop more detailed and specific AMs.

During the period of 6–11 years of age, autobiographical events become longer, more detailed, organized, and coherent and are more memorable in the long term (Bauer & Larkina, 2019; Bauer et al., 2019; Reese & Robertson, 2019). Over childhood, the amount of information children include nearly doubles in comparison to preschool years (Van Abbema & Bauer, 2005), adding data about the *who*, *what, where, when, why,* and *how* of events (Bauer & Larkina, 2019). These advances occur thanks to a series of neurobiological, linguistic, cognitive, and social acquisitions involved in improving memory and that are perfected throughout childhood (Bauer, 2015; Howe, 2014). During this period, language allows individuals to organize, situate and construct personal experience, underlying children’s capacity to understand their own thoughts and emotions and those of others, forming the basis of what will become their autobiographical awareness (Nelson & Fivush, 2020). Children with better language skills at the time of an event are processing that event at a deeper level, which would make it easier to verbally express that memory later (Wang, 2021).

Provision of more specific details about the place and time of events, as well as the ability to correctly order them chronologically, does not occur until early adolescence (Reese, 2014). Adolescence is a period of significant development, including changes in AM (Fivush et al., 2011). Adolescents’ growing capacity to take different perspectives and use abstract reasoning makes them more able to situate specific experiences in the past and link them to the present (Fivush, 2011). Prior to adolescence, children are more likely to focus on single events in their autobiographical recall (Koch & Wang, 2022), and it is not until about 12 years that they begin to link individual events to create a casual narrative (Habermas & de Silveira, 2008).

There are AM-related abilities that appear later in adolescence, as they improve concurrently with neurodevelopment, particularly between the ages of 12 and 16, but continue to mature until the early 20s (Bosmans et al., 2013; Given-Wilson et al., 2018). To provide a "complete" autobiographical recollection, understanding is necessary in three domains: (1) *temporal coherence*, which refers to the ability to organize isolated autobiographical events into a series of organized events, explaining how earlier events influence later ones; (2) *thematic coherence*, which involves organization of a set of meaning in and through an event; and (3) *causal coherence*, which implies the description of the reasons or causes of the events (Given-Wilson et al., 2018; Habermas & de Silveira, 2008). This acquisitions imply narrative skills to integrate single significant past events with present goals and future perspectives (Bartoli & Smorti, 2019). Previous research has shown that this skills allow the child to create a personal life story that can be shared in a social environment (Bluck & Alea, 2011; Conway & Pleydell-Pearce, 2000). Personal life story consists of highly significant personal events that were experienced in the past and are linked to the person’s present and future goals through narrative tools, creating a senses of continuity of the self (Nelson & Fivush, 2004).

Therefore, the retrieval of AMs involves the activation of an extensive brain network responsible of the different cognitive processes engaged during AM retrieval, including language processing (Fossati, 2022; Greenberg & Rubin, 2003). Individual differences in these cognitive abilities may contribute to the observed inter-individual differences in the retrieval of AMs (Palombo et al., 2018). In fact, these differences emerge in early childhood, affecting the later ability to retrieve specific memories (Jack et al., 2009). Thus, Reese et al. (2010) found that adolescents with stronger concurrent language skills recalled more voluminous early memories and those who had stronger receptive language skills in early childhood recalled a greater number of specific memories. Finally, it is worth noting that individual differences in language skills decline once the basic skills have been mastered and are less important during later childhood and adolescence (Fivush et al., 2006).

## The current study

In view of the above, it can be concluded that AM specificity is a mechanism of emotional and cognitive protection (Williams et al., 2007), the development of which is the result of the influence and interrelationships of sociocultural and cognitive factors from preschool age (Nelson & Fivush, 2020). Accordingly, it is important to understand the evolution of this capacity over time, which could help identify ways of enhancing AM to establish preventive strategies through early intervention form emotional and cognitive problems.

The main objective of this longitudinal study, with two repeated measurements, was to analyze the development of AM in a sample of Spanish participants at ages 5 and 12. Available cross-sectional studies on AM cannot report longitudinal changes in AM specificity during childhood. Therefore, we intended to obtain an overview of the evolution of AM from the preschool years to early adolescence applying standardized tests to assess AM in the same sample, with a 6-to-7-year interval. Additionally, it was intended to explore the relationship between the AM and a measure of language at the two time points. To analyze the data, we used a repeated measures study design, with multivariate statistics.

Drawing on previous findings suggesting enhancements in AM specificity from preschool age, we propose the following hypotheses:

I. Considering improvements in AM specificity are directly age-related (e.g., Nieto et al., 2017, 2018), we expect to find a higher number of specific memories in the second measurement.
II. We expect that verbal capacity has an indirect effect in the relationship between age and autobiographical specificity in the first measurement, but not in the second. As previously stated, although language is considered a contributor to the emergence of AMs (Reese, 2002), once basic command of language is acquired, linguistic differences impact much less on individual differences in AM specificity in children (Fivush et al., 2006).

## Method

Data collection took place at two points in time. At Time 1 (T1), 100 children participated (*M* = 62.43 months, *SD* = 6.91; age range: 50–74 months; 56% girls). At Time 2 (T2), 78 children remained in the sample (*M* = 142.71 months, *SD* = 6.59; age range: 132–155 months; 53.8% girls). The reduced sample size at the second measurement was mainly due to the change from primary to secondary school, making it difficult to follow up with all the children. All the children were recruited from a grant-aided school in Spain. All the children were Caucasian Spanish speakers from middle-high socioeconomic status families (yearly income between €25,000 and €65,000). The inclusion criteria were as follows: (1) participant evaluated at the two study time points; (2) absence of psychopathology or learning difficulties; and (3) informed consent by parents or legal guardians. In order to stablish our statistical approach regarding missing data, we tested if there were differences between the sample retained and lost at Time 2 for the main variables measured: age, autobiographical memory, and vocabulary. Results indicated no statistical differences for any of the variables (all *p*s > .05). Additionally, we checked that our data set were missing completely at random using the EM algorithm (Chi^2^ = 5.16; *p* = .08). However, as indicated by Hair et al. (2014) when missing data goes beyond 15% (as in our case, which is 22%), listwise deletion is a better choice to ensure a correct data analysis. In line with this, 22 children were excluded from the study.

## Measures Time 1

### Autobiographical memory test-preschoolers

AM specificity was measured using the Autobiographical Memory Test for Preschoolers (Nieto et al., 2017), a task based on the original procedure designed by Williams and Broadbent (1986). The AMT-P consists of 10 cue words, of which five are positively valenced (*happy*, *loving*, *be friends*, *share*, and *play*) and five are negatively valenced (*sad*, *take away*, *angry*, *argue*, and *hit*). To adapt the task to preschoolers, each word is presented in association with a pictogram. The words and pictograms are presented in a set order, alternating between positive and negative cues. Participants are asked to verbally recall a specific memory to each word. The concept of specificity is previously explained by the use of examples. All participants are given two practice words (*bike* and *story*) to ensure understanding. Following the practice words, the task begins with the presentation of the first cue and its corresponding image. After each cue, the children are given the following instruction: “Think about a specific time when you felt/were/had … and tell me what happened.” They have a minute to answer. In no case may the experimenter help the children to retrieve their memories. If no response to the cue word is generated after the regulated minute, the task goes on to the next word. The memories are coded as specific when they refer to a specific place and moment of time and they last less than a day (e.g., “The day I was given a ball”). Events that are repeated over a certain period of time (e.g., “When I go to my cousins’ house to play”) are coded as categoric memories, and those that last longer than 24 hours (e.g., “We were on holiday at the beach”) as extended memories. All specific, categorical, or extended memories can be coded as positive, negative, or neutral according to its emotional valence. When children relate the cue with another word (e.g., the name of a person, an object) that is considered meaningful, but that does not represent a memory, it is coded as semantic association. Finally, all responses that cannot be coded in any other category are considered nonmemories. In our case, the memories were coded by two separate examiners. Interexaminer reliability for memories was obtained from 51 transcriptions. The final percentage was 94%, with a Cohen’s Kappa coefficient of 0.85.

### Vocabulary

To measure verbal ability, we individually administered the Spanish adaptation of the vocabulary subtest from the WPPSI-III. This test assesses formation of verbal concepts and level of word comprehension. It also includes aptitudes related to general knowledge, learning capacity, and language development. The task comprises 25 items and involves drawing common objects (e.g., candy) and abstract (e.g., polite). Each item can obtain a score from 0 to 2 points. The scores for each item are summed to obtain a direct score, being able to obtain a maximum of 50 points. Reliability for the vocabulary subtest is α = .76.

## Measures Time 2

### Autobiographical memory test

At the second measurement, AM specificity was assessed using the Autobiographical Memory Test by Williams and Broadbent (1986) in the written version, which has showed good consistency with the oral version (Raes et al., 2006). The AMT has been used before in children and adolescents (e.g., Lam et al., 2022; Warne et al., 2020) and its psychometric properties have been validated for the Spanish population (Ros et al., 2018). This test consists in presenting 10 cue words, of which half are positively valenced (*happiness*, *friendship*, *excitement*, *energy*, and *smile*) and the other half negatively valenced (*guilty*, *failure*, *worry*, *sadness*, and *illness*), which are alternately presented. Before beginning the task, the concept of specific memory was explained, and two practice words were administered to ensure understanding. Subsequently, the participants were asked to write down a memory for each cue. No time constraint was imposed. The memory categories were coded in the same way as In the AMT-P. To calculate the percentage of inter-rater agreement on the coding of the AMT, two researchers independently evaluated 100 randomly selected memories. The final percentage was 93%, with a Kappa coefficient of 0.86.

### Vocabulary

To measure the capacity for verbal comprehension and concept formation, we used the vocabulary subtest from the Spanish version of the Wechsler Intelligence Scale for Children (WISC-V; Wechsler, 2015). The test comprises 36 words of increasing difficulty, including common concepts (e.g., *island*) and other more abstract notions (e.g., *rivalry*). The participants are asked to define as precisely as possible the words the examiner reads aloud. Each item can obtain a score from 0 to 2, depending on the accuracy of the definition provided. According to the test instructions, children 11 years old or older start at Item 9. Therefore, in our sample the maximum possible score is 54 points.

It is worth noting that both Wechsler scales (WPSSI-III and WISC-V) are designed to measure the same aspects of verbal ability, although each is adapted to the developmental stage of the participants. Niileksela and Reynolds (2019) conducted a study to investigate whether the constructs measured across these scales are the same. Results suggested that the constructs measured by the Wechsler scales are generally the same and remarkably consistent across different versions, including the WPPSI-III and WISC-V, so the different Wechsler batteries can likely be interpreted similarly.

### Procedure

The Clinical Research Ethics Committee approved the study protocol (reference number: *Record* Nº 2019/01/002), which was also approved by the director and board of governor of the school. This work was conducted over two data collection times (T1 and T2)—that is, the participants were assessed at age 4 and 12. To conduct the study, we obtained the informed consent of both collaborating school management team and the children’s parents/legal guardians at both T1 and T2. At the first measurement, the participants we recruited from among the children enrolled in the 4- and 5-year-old classes of Infant Education. These same children were either in Year 6 of Primary Education or Year 1 of Secondary Education at the time of the second measurement. All the tests were administered during normal school hours and within the school facilities. At T1, the school counsellor administered the AMT-P in a single session and in a separate school room. At the end of the individual assessment session, the person tasked with data collection accompanied the children back to class. At the second measurement, the AMT was administered to all children in each year group together.

### Data analysis

Our statistical analyses were conducted using IBM SPSS Statistics 24 (SPSS, Inc., Chicago, IL). The criterion for statistical significance was set at *p* ≤ .05. First, we checked that the variables conformed to the normal distribution using the Kolmogorov–Smirnov test and graphical procedures, and homoscedasticity was verified for the comparison of groups, using the Levene test (all *p*s > .05). Second, we conducted the descriptive analysis of the main study variables and conducted the *t* test for independent samples to determine any possible gender-related statistical differences. Descriptive analyses were conducted for all the study variables (T1 and T2) but including only the children for whom we had data from the two measurements. Thirdly, we calculated Pearson correlations between the domains of AMT and between these domains and vocabulary scores at each measurement. Fourthly, we conducted simple regression analyses to examine whether age and vocabulary contributed to AM specificity at both times. Fifth, we used Student’s *t* test for paired samples to analyze the mean differences of the AM tests and vocabulary scores between the first and second measurement. Finally, to examine the possible indirect effect of vocabulary in the relationship between age and AM specificity at both measurement moments, we applied Model 4 in PROCESS, with a 95% confidence interval and 10000 bootstrapping samples.

## Results

### Descriptive results

Means and standard deviations were calculated for both total scores of each domain of the AMT (Specific Memory, Extended Memory, Categorical Memory, Semantic Association and No-Memory) and for positive, negative, and neutral memories within each domain, as well as for vocabulary scores at the two measurement moments. Table 1 shows the results for the total sample of participants and by gender. In addition, a *t* test was calculated to control for significant gender-related differences for all the variables analyzed in the study sample. The results revealed no statistically significant differences between boys and girls in the main variables (all *p*s > .05). Descriptive results for the AMT showed that the mean number of specific memories increased with age (total, positive, and negative), as well as the number of extended memories. The trend was inverse in the case of categorical memories, sematic associations and no-memories. Regarding the vocabulary task, the results revealed an increase in the verbal capacity scores.

**Table 1:** Descriptive results and analysis of the differences between measurements (T1 and T2) in the variables studied

	T1		T2		Student’s Test Paired-Samples (T1-T2)				
	*N* = 100		*N* = 78						
Domains	*M*	*SD*	*M*	*SD*	*t* (*df*)	*p*	Mean difference T1-T2	*SD*	Cohen’s *d*
Total specific memory	5.22	2.46	7.04	1.98	−6.31*** (77)	.001	−1.97	2.74	−.72*
Positive specific memory	2.44	1.25	3.62	1.25	−6.76*** (77)	.001	−1.27	1.65	−.77*
Negative specific memory	2.78	1.69	3.40	1.27	−3.10*** (77)	.001	−.66	1.87	−.35*
Neutral specific memory	.01	.10	.04	0.95	−1.75* (77)	.04	−.05	.19	−.20
Total extended memory	.51	.69	1.42	1.18	−6.00*** (77)	.001	−.86	1.25	−.68*
Positive extended memory	.39	.58	.40	0.59	.44 (77)	.33	.04	.78	.05
Negative extended memory	.12	.32	.99	.95	−7.70*** (77)	.001	−.87	.99	−.87*
Neutral extended memory	.00	.00	.03	.16	−1.42 (77)	.08	−.02	.16	−.16
Total categorical memory	2.00	1.74	.83	1.52	4.42*** (77)	.001	1.17	2.31	.51*
Positive categorical memory	1.27	1.03	.60	1.08	4.33*** (77)	.001	.71	1.43	.50*
Negative categorical memory	.73	1.06	.22	.60	3.58*** (77)	.001	.48	1.17	.41*
Neutral categorical memory	.01	.10	.01	.11	−1.00 (77)	.16	−.01	.11	−.11
Semantic association	.51	.76	.01	.11	6.10*** (77)	.001	.56	.80	.69*
No memory	1.75	2.04	.71	1.23	3.98*** (77)	.001	1.06	2.34	.45*
Vocabulary (a)	19.25	5.93	43.50	5.45	.07 (77)	.47	.01	1.15	.01

*** *p* < .001; **p < .01; *p < .05; (a) For the analysis of the differences in the vocabulary measures between T1 and T2, the *z*-scores were used

### Correlation analysis

Within each time measurement, T1 and T2, and between them, we calculated the correlations for the total scores of the AMT domains and between the AMT and vocabulary scores. The results can be seen in Table 2. Within T1, the specificity of AM calculated using the AMT-P was negatively correlated with categorical memories, semantic associations and nonmemories (all *p*s < .01), while it was positively associated with vocabulary scores (*p *< .01). Within T2, the specificity of AM calculated using the AMT was negatively associated with categorical and extended memories (*p*s < .01) and with non-memories (*p *< .05); we did not find a significant correlation between AM specificity and vocabulary scores at T2. Additionally, specificity at T1 was positively associated with specificity (*p* < .05) and vocabulary (*p* < .01) at T2; in turn, it correlated negatively with the Categorical Memory and Semantic Association domains at T2. Finally, a positive correlation was established between the vocabulary scores at T1 and T2 (*p* < .01).

**Table 2:** Correlations between main variables (T1 and T2)

	1	2	3	4	5	6	7	8	9	10	11	12
1. T1 Total Specific Memory	–											
2. T1 Total Extended Memory	−.09	–										
3. T1 Total Categorical Memory	−.48**	−.05	–									
4. T1 Semantic Association	−.32**	−.09	.00	–								
5. T1 No Memory	−.61**	−.15	−.28**	−.03	–							
6. T2 Total Specific Memory	.27*	−.07	.01	−.28*	−.19	–						
7. T2 Total Extended Memory	.04	.20	−.14	−04	−0.3	−.48**	–					
8. T2. Total Categorical Memory	−.25*	−.17	−.03	.24*	.29*	−.60**	−.05	–				
9. T2 Semantic Association	−.24*	−.09	.07	.06	.25*	−.06	−.14	−.06	–			
10. T2 No Memory	−.13	.14	.15	.10	−.05	−.41*	−.11	−.20	.21	–		
11. T1 Vocabulary WPPSI-III	.60**	.03	−.26*	−.30**	−.38**	.07	.08	−.09	−.12	−.043	–	
12. T2 Vocabulary WISC-V	.29**	.19	−.13	−.17	−.28*	.22	−.11	−.22	−.05	.02	.34**	–

*N* = 78; **p < .01; *p < .05

### Simple regression analysis

To examine whether age and actigraphic sleep measures vocabulary scores contributed to specificity of the AM during preschool years and early adolescence, we performed hierarchical regression analyses. At Time 1, according to regression models age explained approximately 22% of the variance in AM specificity when included alone (Adjusted *R*^2^ = .217), *F*(1, 98) = 28.51, *p* < .001, and age and vocabulary together explained approximately 47% of the variance (Adjusted *R*^2^ = .473), *F*(2, 97) = 45.51, *p* < .001. Both age ( β = .36), *t*(77) = 4.78, *p* < .001, and vocabulary scores (β = .52), *t*(77) = 6.98, *p* < .001, were significant predictor of AM specificity at Time 1. In contrast, at Time 2, the regression model was not significant, either when age was introduced in Step 1 (Adjusted *R*^2^ = .01), *F*(1, 75) = 0.08, *p* = .78, or when age and vocabulary were included in Step 2 (Adjusted *R*^2^ = .05), *F*(1, 75) = 1.95, *p* = .15. Therefore, age and vocabulary scores were not significant predictors of AM specificity at Time 2.

## Student’s paired-samples test (T1–T2)

The results of the analyses of the mean differences between the T1 and T2 measurements in the AMT and the vocabulary scores can be seen in Table 1. There were significant differences between the mean scores at the two measurements in all the total domains of the AMT: Total Specific Memory, Total Extended Memory, Total Categorical Memory, Semantic Association and No-Memory. The Total Specific Memory domain, *t*(77) = −6.31, *p* < .001, presented a lower mean score at the T1 (*M* = 5.22, *SD* = 2.46) than at the T2 (*M* = 7.04, *SD* = 1.04); the same was found for the Total Extended Memory domain, *t*(77) = −6.00, *p* < .001, *M* difference = −.86. In contrast, *t* analysis showed higher mean scores at the T1 than at the T2 for the following domains: Total Categorical Memory, *t*(77) = 4.42, *p* < .001, *M* difference = 1.17; Semantic Association, *t*(77) = 6.10, *p* < .001, *M* difference = .56; and No-Memory, *t*(77) = 3.98, *p* < .001, *M* difference = 1.06. Finally, no differences were found between the mean vocabulary scores between T1 and T2.

**Fig. 1:**
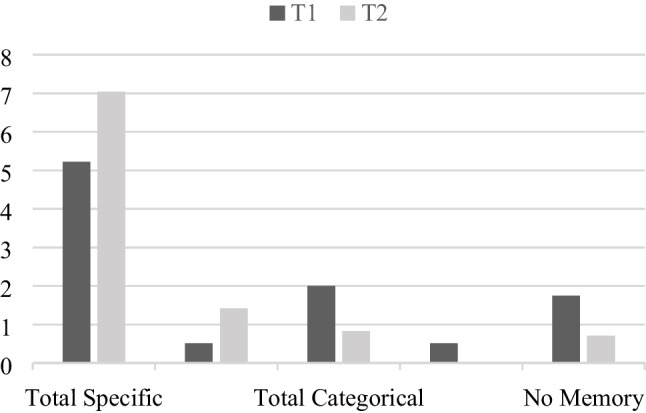
Mean scores obtained at the first measurement (T1) and the second measurement (T2) in the Autobiographical Memory Test. *Note.* All *p*s < .001

Figure 1 showed a graphic representation of the main scores obtained at the first and second measurements for the AMT.

### Tests of indirect effects

Test of indirect effects were performed to assess the indirect role of Vocabulary in the relationship between Age and Total Specific Memory at both temporal moments.

**Fig. 2:**
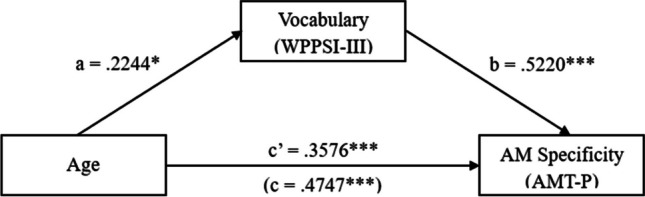
Mediation results at T1. Regression standardized coefficients (a, b, c, and c′) for the relationship Between Age and AM Specificity as partially mediated by vocabulary. *Note. N* = 78; *** *p* < .001; **p < .01; *p < .05; WPPSI: Wechsler Preschool and Primary Scale of Intelligence; AMT: Autobiographical Memory Test for Preschoolers

At T1, as shown in Fig. 2, the test revealed that (a) age was associated with performance of the vocabulary task (Path a), β = .2244, *t*(77) = 2.28, *p* = .03; (b) performance on the vocabulary task was associated with AM specificity (Path b), β = .5220, *t*(77) = 6.97, *p* < .001; (c) the direct effect of age on AM specificity was significant (Path c’), β = .3576, *t*(77) = 4.77, *p* < .001; and (d) the indirect effect of age on AM specificity through vocabulary was also significant (β = .1171, 95% CI [.0191, .2203]). Hence, we found an indirect effect of vocabulary in the relationship between age and AM specificity at T1.

At T2, we did not find an indirect effect of vocabulary in the relationship between age and AM specificity. In this case, age was not associated with either the specificity of AM, β = .0315, *t*(77) = 0.28, *p* = .79, or the vocabulary score, β = .0102, *t*(77) = 0.89, *p* = .92. Performance on the vocabulary task was not associated with AM specificity, although the results are close to statistical significance, β = .2219, *t*(77) = 1.95, *p* = .05.

## Discussion

The main objective of the present study was to examine the development of children’s AM across a period of time and its relationship with verbal functioning. We focus on two main aims: (1) to analyze the evolution of specific memories by taking measurements when participants were in preschool (Time 1) and, later, in the last year of primary school or at the beginning of secondary school (Time 2); (2) to examine the influence of verbal abilities on specific recall, and to understand whether verbal capacity has an indirect effect in the relationship between age and autobiographical specificity at both time points.

Concerning the first aim, we found that, with age, there is an increase in the number of specific memories and, to a lesser extent, extended memories, while the number of categorical memories, semantic associations and no memories decrease. This is consistent with previous research that has suggested children represent and retrieve memories of a general nature from early childhood, and that AM specificity improves with age (Fivush, 2011; Nieto et al., 2018). This development reaches a certain stability during adolescence, when the ability to create a detailed and well-structured narrative of past experience allows adolescents to build a coherent life story (Heron et al., 2012). Most of the available research has concentrated on early childhood, with less attention focused on the development of AM from the preschool years onward (Bauer & Larkina, 2019).

Our results show a significant number of categorical memories during the preschool period, which would be in line with studies that suggest the tendency of infants to generalize the information obtained from their experiences to other domains or events, in addition to their difficulty in integrating the details of events at a specific time and place (Nelson & Fivush, 2004; Newcombe et al., 2007). Therefore, although from very early ages infants are able to use their personal experiences to talk about past events, they are not yet fully capable of spontaneously remembering or reexperiencing specific autobiographical events (Willoughby et al., 2012). Furthermore, from a maturational perspective, different studies have suggested that the apparent difficulty of preschoolers in retrieving specific AMs is due to immature functioning of the hippocampus, a brain structure necessary for the successful encoding and retrieval of AM (Audrain et al., 2022; Barry et al., 2021).

We also found an increase in the number of specific memories when the sample were in their early adolescence. Previous studies have shown gradual age-related increases in AM specificity across childhood (Picard et al., 2009; Piolino et al., 2007). Moreover, it seems that AM continues to develop beyond adolescence (Koch & Wang, 2022). As adolescence is reached, the number of memories and the amount of detail reported for past events increase (Bauer et al., 2016) and the organization of autobiographical narratives improves (Reese et al., 2011). However, the ability to situate AMs at a specific time and place begins to develop in the early years of adolescence and continues to do so throughout adolescence, so a decline in extended memories in favour of specific ones would be expected as the adolescent grows and develops these cognitive abilities (Willoughby et al., 2012). This increase in the number, solidity and complexity of AMs appears to be mediated not only by biological and cognitive variables but also by sociocultural factors (Markowitsch & Welzer, 2010).

Our second aim focused on the relationship between language variables and AM specificity at the two points of time, as well as on the indirect effect of language between age and MA. We found a positive correlation between the AMT and vocabulary during the preschool years, but these relationship was no longer significant at the second measurement. In addition, in the first measurement, significant negative correlations were found suggesting that a higher vocabulary score is associated with a lower probability of categorical memories, semantic associations, and non-memories. This suggest that, while linguistic competence is shown to be a key predictor of AM specificity in early childhood and a source of individual differences in the ability to use language to narrate personal events, these differences disappear once a certain basic command of language has been reached, and this competence is no longer an important predictor of the capacity to retrieve specific memories (Fivush et al., 2006). Language acquisition in early childhood modifies children’s capacity to understand their inner lives, thoughts and emotions, as well as those of others, forming the basis of that will become their autobiographical consciousness (Nelson & Fivush, 2020). From a sociocultural perspective, it has been suggested that an elaborative narrative style in families during early childhood encourages the development of specific AM skills, which help children organize and elaborate their personal experiences (Fivush, 2019). Mother–child reminiscing conversations have a fundamental role in facilitating the social-emotional and cognitive functioning of children (Edler et al., 2021). In fact, Valentino et al. (2021) showed, through a longitudinal study, that the use of an intervention designed to improve maternal elaboration and sensitive guidance during reminiscence could be positively related to the specificity of children’s AM over time. This suggest that language may indirectly affect such specificity during preschool years.

However, the role of language on memory decreases with age. It is likely that once the child has attained the basic language skills and vocabulary necessary to express, and even encode, their memories, these language differences cease to have a critical influence on autobiographical recollection (Reese, 2009). Thus, in our sample no significant correlations were found between language scores and the specificity of AM when the participants were in early adolescence. This is consistent with previous research. By the end of preschool years, and continuing through adolescence, children’s language skills at the moment of measurement are no longer correlated with their verbal memories (Cleveland & Reese, 2008; Jack et al., 2009), nor is their language skills strongly correlated with the coherence of their autobiographical narratives (Reese et al., 2011). Moreover, a positive and significant correlation was found between vocabulary scores in preschool age and early adolescence, while no significant differences were found when longitudinally comparing vocabulary scores between the two measures, suggesting that language skills are remain more or less stable over time. In this senses, the findings of a recent review provide evidence that those children with a more elaborate language, due to a high elaborative maternal reminiscing, was associated with children’s ability to provide greater detailed personal memory, both concurrently and longitudinally (Wu & Jobson, 2019). These results may suggest that those children with a better language ability in preschool will maintain this language proficiency at later ages, affecting the detail and specificity of autobiographical recollection.

Finally, to determine more concretely whether performance on the language tasks has an indirect effect in the relationship between age and the specificity of AM, two test of indirect effects were conducted, one at each point in time. As hypothesized, vocabulary scores appear to have an indirect effect in the relationship between age and autobiographical specificity when children are in the preschool years, but not later. It seems that by the time children enter primary school (at about 6 years of age), they all possess the basic language that allows them to remember events verbally, and vocabulary is no longer a delimiting factor for those memories (Reese, 2009). However, in most studies with older children and adolescents language has been measured only in terms of vocabulary. It is likely that the dimensions of language that may be affecting AM change with age, since it is narrative coherence, and not vocabulary, that undergoes the most changes during adolescence (Given-Wilson et al., 2018). Therefore, it would be interesting for future research with older children to analyze whether higher order language skills may continue to affect the specificity of AM.

This work has a series of limitations. The first is related to the characteristics of the AMT itself. In this sense, there has been some discussion on whether the AMT actually measures autobiographical specificity or merely reflects a specific or general response style when exposed to certain cue words (Griffith et al., 2012; Heron et al., 2012). This limitation has been underlined in both preschool samples, using the AMT-P (Nieto et al., 2017), and in samples of adolescents where the original version of the test was used (Heron et al., 2012). Additionally, following Ros et al. (2018), there is currently no standardized set of cue words for the AMT, and hence the use of different words might arguably lead to differing results. Furthermore, it should be considered that for the present study the two different versions of the AMT were used, and that the preschool version was individually administered while the adolescent version was administered in group format. The second limitation lies in the selection of participants. We used a convenience sample of Caucasian children from middle-high socioeconomic status families, and without any diagnosed psychopathologies, which could affect the extrapolation of our findings to other settings. In these sense, has been suggested that AM may be influenced by cultural orientation (Wang, 2021), and children from Western cultures, like those in our study, might produce more specific memories that those in Eastern societies (Fivush, 2011). Certain cultural elements (e.g., beliefs, cognitive styles, or the importance given to the self and social group) could affect how parents—and mothers, particularly—share information about past memories with their children (Wang, 2021). More specifically, according to Wang (2021), Western mothers offer more details about past experiences when communicating with their children, focus more on their own individuality and autonomy and give more importance to emotional expression; in contrast, in Asian cultures, the affiliation of individuals with their group and common goals are prioritized, and often emphasize children’s behavioural standards and discipline. As a consequence, Western children tend to provide more specific details of past events (e.g., Schröder et al., 2012), and are more focused on their unique attributes, emotions and qualities (e.g., Wang, 2006) than their non-Western peers. Consequently, it would be interesting to replicate this research with samples from other cultures. In addition, for future work it would be interesting to include maternal reminiscence style, which research seems to indicate contributes to individual differences in preschoolers’ autobiographical recall, both longitudinally and cross-sectionally (Wu & Jobson, 2019). Thus, more exhaustive studies are needed, comparing short age intervals or using different techniques to analyze AM (Guler & Mackovichova, 2019). Moreover, further research is needed on changes in AM and their relationship with verbal variables across childhood and adolescence, a period that has been the subject of relatively limited attention in the literature (Bauer & Larkina, 2019).

In conclusion, this work provides new data on the development of AM over the years and on the predictor role of language on AM specificity in preschool and early adolescence. We consider that more research is needed on this topic for several reasons: (a) because research on the development of AM has focused in the emergence of these skills during preschool years and we know relatively little about its development in childhood and adolescence (Bauer & Larkina, 2019); (b) from a clinical perspective, considering that difficulties in recovering specific AMs have been linked to certain psychopathological disorders such as depression (Barry et al., 2021), the study of the evolution of AMs can contribute to the development of new nonintrusive techniques that protect children and adolescents against the appearance of emotional disorders (Pile et al., 2021); and (c) because the early detection of deficits in specificity and language skills could favour the development of school programmes for the prevention of emotional and/or cognitive risks, difficulties in adapting to the environment or lack of self-regulation from an early age (Nieto et al., 2019).

## Data Availability

Availability of data are available on request from the authors. The data that support the findings of this study are available from the corresponding author upon reasonable request.
